# Wearable Sensors and the Assessment of Frailty among Vulnerable Older Adults: An Observational Cohort Study

**DOI:** 10.3390/s18051336

**Published:** 2018-04-26

**Authors:** Javad Razjouyan, Aanand D. Naik, Molly J. Horstman, Mark E. Kunik, Mona Amirmazaheri, He Zhou, Amir Sharafkhaneh, Bijan Najafi

**Affiliations:** 1 VA HSR&D, Center for Innovations in Quality, Effectiveness and Safety, Michael E. DeBakey VA Medical Center, Houston, TX 77030, USA; javad.razjouyan@va.gov (J.R.); anaik@bcm.edu (A.D.N.); mhorstma@bcm.edu (M.J.H.); mkunik@bcm.edu (M.E.K.); 2 Interdisciplinary Consortium on Advanced Motion Performance (iCAMP), Division of Vascular Surgery and Endovascular Therapy, Michael E. DeBakey Department of Surgery, Baylor College of Medicine, One Baylor Plaza, MS: BCM390, Houston, TX 77030, USA; Mona Amirmazaheri@bcm.edu (M.A.); he.zhou2@bcm.edu (H.Z.); 3 Department of Medicine, Baylor College of Medicine, Houston, TX 77030, USA; amirs@bcm.edu; 4 South Central Mental Illness Research, Education and Clinical Center (MIRECC), Houston, TX 77030, USA

**Keywords:** frailty, pre-frail, wearable sensor, physical activity, sedentary behavior, moderate-to-vigorous activity, steps

## Abstract

*Background:* The geriatric syndrome of frailty is one of the greatest challenges facing the U.S. aging population. Frailty in older adults is associated with higher adverse outcomes, such as mortality and hospitalization. Identifying precise early indicators of pre-frailty and measures of specific frailty components are of key importance to enable targeted interventions and remediation. We hypothesize that sensor-derived parameters, measured by a pendant accelerometer device in the home setting, are sensitive to identifying pre-frailty. *Methods:* Using the Fried frailty phenotype criteria, 153 community-dwelling, ambulatory older adults were classified as pre-frail (51%), frail (22%), or non-frail (27%). A pendant sensor was used to monitor the at home physical activity, using a chest acceleration over 48 h. An algorithm was developed to quantify physical activity pattern (PAP), physical activity behavior (PAB), and sleep quality parameters. Statistically significant parameters were selected to discriminate the pre-frail from frail and non-frail adults. *Results:* The stepping parameters, walking parameters, PAB parameters (sedentary and moderate-to-vigorous activity), and the combined parameters reached and area under the curve of 0.87, 0.85, 0.85, and 0.88, respectively, for identifying pre-frail adults. No sleep parameters discriminated the pre-frail from the rest of the adults. *Conclusions:* This study demonstrates that a pendant sensor can identify pre-frailty via daily home monitoring. These findings may open new opportunities in order to remotely measure and track frailty via telehealth technologies.

## 1. Introduction

According to a 2014 United States Census Bureau report, the population aged 65 and over has been projected to grow from 43.1 million in 2012 to 83.7 million in 2050 [1]. One of the distinctive health states related to the aging process is frailty [2]. The prevalence of frailty in the ambulatory population is about 15% and the prevalence of pre-frailty is about 45% [3]. Frailty places older adults at risk for dramatic changes in physical and mental well-being following challenges to their health, such as an infection, injury, or medication interactions [4]. Frailty is an independent predictor of adverse outcomes, including falls, delirium, hospitalization, and mortality [5]. Identifying patients at risk for frailty (pre-frail adults) would enable healthcare providers to intervene from an early stage so as to mitigate some of the potential adverse sequelae [6].

Although there is no consensus on the definition of frailty, recent efforts have focused on a standardization of the definition so as to enhance its application in clinical care [7]. Geriatricians used to say, “I know it when I see it, but what I see may not be the same as what everyone else sees” [8]. The definition of frailty has evolved from the stereotypical description of a “thin, stooped, slow octogenarian” person [9]. Approaches introduced by Fried [10] and Rockwood [11] have had the strongest empirical and conceptual support.

Fried and colleagues developed a frailty phenotype theory based on mutually exacerbating cycles of negative energy balance, sarcopenia, and diminished strength and tolerance for exertion in the community dwelling geriatric population [7]. In this theory, the frailty phenotype is associated with declining energy and reserve [10]. Fried proposed five core clinical criteria for the impairment that is underlying frailty, namely, shrinking, exhaustion, inactivity, slowness, and weakness [10]. Older adults are classified as frail, pre-frail, or robust. An individual meeting the threshold of impairment for three of these criteria are classified as frail. The individual meeting criteria for one or two components is classified as pre-frail. Those meeting none of the impairment criteria are classified as non-frail or robust. Individuals meeting the pre-frail criteria can potentially benefit more from clinical intervention [12] compared with those meeting the frail and non-frail (robust) criteria [13]. Furthermore, a greater variety of interventions are potentially available to pre-frail older adults, who require less supervision than frail older adults [14].

The Fried frailty phenotype has several diagnostic limitations. The Fried approach has been described as impractical in a busy clinic settings [15], not designed for inpatient or bed-bound older patients [16], not sensitive to subtle physiological changes [17], and as failing to account for the important domain of cognition function [18]. Additionally, the approach’s reliance on questionnaires to identify weight loss, exhaustion, and energy expenditure suffers from participant bias [19,20,21,22].

To overcome limitations of the Fried frailty phenotype, researchers have proposed wearable sensors as an alternative to assessing the frailty phenotype [15,23,24,25]. These wearable sensors can address the challenges in measuring frailty, such as feasibility, practicality, ease of use, accessibility, reproducibility, and reliability, without hindering daily activity in the outpatient or inpatient settings [15,23,24]. Previous studies using a wrist sensor, by Lee et al., have demonstrated that a 20-s upper extremity test is capable of predicting frailty in the outpatient setting [26] and in community dwelling settings [27]. Schwenk et al. have shown that multiple sensor-based physical activity monitors, which measure posture (walking, standing, sitting, lying, and postural transition from sit-to-stand and stand-to-sit) and gait parameters (stride length, gait speed, gait velocity, and cadence), are capable of discriminating between non-frail, pre-frail, and frail patients [24]. Other studies have shown that sensor-derived activity levels (sedentary behaviors, light and moderate-to-vigorous activity) have a high correlation with frailty status [28] and are capable of discriminating between different frailty statuses [29,30,31,32]. They found that an increase in the sedentary behavior and a decrease in the high intensity activity, such as moderate-to-vigorous activity, is a strong predictor of frailty progression. Interestingly, Theou et al. showed that a single parameter, the number of steps, which is derived from a wearable sensor is significantly correlated with the progression of frailty [31]. Furthermore, studies of sensor-based in-home sleep monitors have found an association between sleep disruptions and [33] the existence of frailty [34].

While few studies proposed daily step measurement for in-place monitoring frailty status, to our knowledge, no prior studies have examined fine-grain characteristics of daily physical activities, such as activity behavior (e.g., sedentary), activity postures (e.g., sitting, standing, lying, and walking), and walking characteristics (e.g., number of taken steps), which are measured by a single senor into a cohesive model. Such models are potentially valuable because they would provide a clinical and technical validation of these sensor-derived parameters, and serve as a basis for future studies developing predictive models of change between frailty categories. In particular, there are very few studies that are enabled to identify pre-frailty using wearable-based activity monitoring. Pre-frailty is considered as the early stage of frailty [35,36,37]. While several studies have suggested that frailty is not an irreversible process, it has been hypothesized that the early detection of a pre-frail status may provide a window of opportunity for timely preventive or therapeutic interventions, which may delay the progression of frailty and even reverse it [35,36,37]. Thus, the early detection of pre-frailty may provide a unique opportunity to provide a timely intervention and is desperately needed. Therefore, the purpose of this study is to examine the ability of a practical wearable platform (a pendant accelerometer), to remotely monitor the frailty stages using daily activity monitoring, with an emphasis on distinguishing pre-frailty. Specifically, our first aim was to determine which sensor-derived parameters—including walking characteristics (e.g., daily number of taken steps); activity patterns, including postures (i.e., sitting, standing, and lying) and walking durations; activity patterns, including sedentary, light, and moderate-to-vigorous activities; and sleep parameters, including total sleep time and sleep efficiency—are capable of discriminating between the three frailty categories. The second aim was to identify the most significant independent parameters in order to discriminate the pre-frail from other groups. Finally, our third aim was to build a composite model that would have a promising performance so as to discriminate the pre-frail stage from non-frail and frail stages.

## 2. Materials and Methods

### 2.1. Participants and Assessment

#### 2.1.1. Participants Recruitment

We recruited ambulatory older adults that were ≥60 years of age, who were able to walk 15 feet (~4.5 m) independently, with or without aid. Participants were enrolled from outpatient clinics or community dwelling settings. Exclusion criteria were severe cognitive impairment (a Mini-Mental State Examination [MMSE] score ≤16) [38] and those unable/unwilling to consent. Participants who met the eligibility criteria signed written consent form. This study was approved by the local institutional review boards.

#### 2.1.2. Demographic and Clinical Characteristics

Trained clinical coordinators collected patient demographic and clinical characteristics. The measures were history of falls, height, weight, and fear of falling, which was assessed by the Fall Efficacy Scale-International (FES-I) [39]. Participants’ depression scale was measured using the Center for Epidemiologic Studies Depression Scale (CES-D) [40].

#### 2.1.3. Frailty Assessment

We used the Fried frailty phenotype assessment to stratify the participants into three groups, namely, non-frail, pre-frail, and frail [10]. The Fried frailty assessment consisted of five phenotypes, namely, shrinking (losing more than 10 lb. in prior year unintentionally), exhaustion (self-reported questionnaire), inactivity (self-reported questionnaire), slowness (prolonged performance during 15-feet walk test), and weakness (decreased grip strength) [10]. If the participants’ performance placed them in the lowest quartile for a phenotype, they received one point for that phenotype. For the final score, the sum of all of the one points (*SUM*) was calculated and the subject was classified into one of three groups:

$$Frailty\;Status=\;\{\begin{array}{cc} nonFrail/Robust:\; & SUM=0\\ preFrail: & 0\;<\;SUM\;\leq 2\\ Frail: & SUM\geq 3 \end{array}$$

### 2.2. Sensor Based Assessment

**Figure 1:**
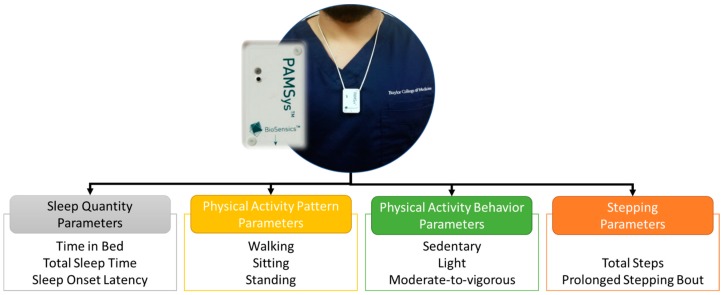
The sensor placement (above) and the common parameters (below) that were extracted, based on validated algorithm, namely: sleep quantity, physical activity patterns, physical activity behaviors, and stepping count.

We used a pendant sensor (PAMSys™, BioSensics LLC, Watertown, MA, USA), which was placed at the sternum (Figure 1). The participants were instructed to keep the sensor on for 48 h and then return it to the center, through either a paid envelope or collection by the study coordinators. The PAMSys had three dimensional accelerations that recorded the gravity and inertial accelerations, with a sampling frequency of 50 Hz. The sensor had a built-in memory that allowed for the saving of data and also downloading it to the computer via the company software that was provided. We used PAMWare™ software (BioSensics, Watertown, MA, USA) to download, calibrate, and normalize (to gravity or g) the data. All of the physical activity and sleep parameters were extracted from the pendant sensor. However, two different validated algorithms were used to extract the physical activities and sleep parameters using chest acceleration, as described in our previous studies [41,42,43,44].

#### 2.2.1. Physical Activity Behavior Parameters

For the purpose of this study, the physical activity behavior parameters that were considered were sedentary behavior (Sed), light activity (Lgt), and moderate-to-vigorous activity (MtV). Sedentary behavior was defined as an activity with less than 1.5 metabolic equivalent (MET), such as sitting or lying [45,46]. Light activity was defined as an activity between ≥1.5 MET and MET <3.0, such as hanging out the washing, ironing and dusting, and working at a standing workstation [46]. Moderate-to-vigorous activity referred to an activity demanding ≥3.0 MET, such as brisk walking, recreational activities, climbing stairs, etc. [46].

To measure the physical activity levels in each category, we calculated the mean amplitude deviation (MAD) [47]. Before we calculated the MAD, several steps were taken. Firstly, we preprocessed the data in order to remove the high frequency activities that had not originated from human body [41]. We used a wavelet filter bank (Daubechies [48]) with a cut-off at 12.5 Hz. The wavelet filter was used, as it has been shown to keep the morphology of signal better than the other filters [49]. Then, we calculated the following:

(1) $$r_{i}=\sqrt{x_{i}^{2}+y_{i}^{2}+z_{i}^{2}}$$

Here, $r_{i}$ is the norm acceleration containing the static and dynamic component of the body accelerations for each sample (*i*). The $\left(x_{i},y_{i},z_{i}\right)$ are the three-dimensional accelerations. For each 6-s epoch, the average $r_{i}$ ($R_{ave}$) of the 300 samples (=6 s × 50 Hz) was calculated as follows:

(2) $$R_{ave}=\frac{1}{N}\sum_{i=1}^{N=6\;s}r_{i}$$

The MAD value for each epoch was calculated as the absolute sum of distance from $R_{ave}$ as follows:

(3) $$\mathrm{MAD}=\frac{1}{N}\sum_{i=1}^{N=300}\left|r_{i}-R_{ave}\right|$$

The MADs for all of the possible epochs were calculated. The unit of MAD is in the milligravity, where 1 g is equal to 1000 mg. We used three cut-points to classify activity level into sedentary (MAD < 20), light (20 ≤ MAD < 90) and moderate-to-vigorous (MtV: MAD ≥ 90). This method had, on average, a very high sensitivity and specificity of 98% and 96%, respectively, in order to detect the physical activity levels [47].

#### 2.2.2. Non-Wear Time and Valid Day of Monitoring

We excluded the intervals when participants did not wear the sensors. These non-wear periods occurred during aquatic activities, such as bathing, or because the participant forgot to wear the sensor. We used a method that was validated in older adults [50], which defined non-wear periods as ≥90 min with no MAD (allowing for 2 interrupted minutes with MAD of <20).

A valid day of monitoring was defined as ≥8 hours of wear [45,50]. We used the valid day with the wear time annotation to report the physical activity parameters. The average of the activity parameters over the 2 days were reported to have reached the highest inter-class correlation (ICC) [51].

For each valid day of monitoring, we calculated the following parameters:

- Total activity: the sum of the all of the specific activity (Sed, Lgt, and MtV).
- Percentage activity: the total activity duration of a specific activity, divided by the total duration of wear time, excluding the nocturnal time in bed.
- Median activity: the 50th percentile of the bout of the specific activity.
- Health and Human Services (HHS) guideline, %: The percentage of participants who met the U.S. Department of HSS recommendations that an adult should have at least 300 min of moderate-to-vigorous activity per week [52]. To calculate this parameter, we estimated those who had at least 42 min (300 min/7 days = ~42 min) of moderate-to-vigorous physical activities per day.

The bout of activity was the consecutive, continuous interval of an activity without any interruption, such as Sed, Lgt, or MtV.

#### 2.2.3. Physical Activity Pattern and Stepping Parameters

The postural parameters that were calculated from the PAMSys sensor’s raw data included lying, sitting, standing, walking, and the number of steps. The algorithm first detected the episodes of walking, which was three consecutive steps with less than specific time intervals [42,43,44,53]. The steps were determined by the peaks in vertical acceleration, where the signal passed through a wavelet-based band pass filter, with absolute values greater than a certain threshold. Standing, sitting, and lying were considered non-walking intervals. Lying intervals were identified when the vertical acceleration was close to zero gravity. In the other words, during the lying intervals, the vertical vector was at a right angle with the frontal plain. Sitting and standing were identified through the pattern changes in frontal-vertical vectors. The sensitivity (87% to 99%) and specificity (87% to 99%) of the algorithm was reported previously [43].

The postural data was reported for each 24 h period and the average was calculated for the final outcomes, as follows:

- Posture, %: the duration of each posture (lying, sitting, standing, walking) in 24 h.
- Total steps: the total number of steps per day.
- Longest unbroken posture, s: the maximum duration of an unbroken bout for each posture.
- Median posture, s: the median duration of a bout for each posture.
- Longest unbroken stepping bout: the number of steps during the longest bout of stepping without interruption.
- Median stepping bout: the number of steps in the median bout of stepping without interruption.

#### 2.2.4. Sleep Quantity Parameters

Using the physical activity algorithm [41,44], the start and end of sleep during night time were recorded in order to estimate the time spent in the bed and out of the bed. The sleep algorithm was applied only during the time in the bed. The method for extracting sleep parameters of interest, using a chest accelerometer, was described in detail in our previous study [41]. In summary, firstly, the acceleration data passed through a band pass filter, then a vector magnitude/norm of acceleration was built and a minute wise signal was calculated. Next, a feature vector, which consisted of an activity intensity in the moment and a standard deviation of the activities as well as any sleep position changes, was built for each minute and fed to a model. Finally, the model estimated the sleep/wake conditions. From the sleep/wake signal, the sleep quantity parameters were extracted as follows:

- Time in bed (TiB), hours: the total duration of a participant’s time in bed.
- Total sleep time (TST), hours: the total duration of nocturnal sleep.
- Sleep onset latency (SOL), min: the total interval of the time to fall asleep, from the beginning of TiB.
- Wake after sleep onset (WASO), min: the total duration of the time awake, after sleep onset until sleep offset.
- Sleep efficiency (SE), %: the percentage of TST to onset of sleep to last offset of sleep.
- Supine, %: the total duration of supine during TiB.
- Prone, %: the total duration of prone during TiB.
- Sides, %: the total duration of side lying (left or right) during TiB.

### 2.3. Statistical Analysis

We used the Fisher exact test to evaluate the differences between the categorical variables (demographic or clinical characteristics). We used the ANCOVA with the Tukey LSD post hoc test, which was performed on the SPSS (IBM, V24.0.0), in order to test the significance level between the three groups of non-frail, pre-frail, and frail. We also estimated the Cohen’s d effect size (*d*), where *d* ≈ 0.2, 0.5, and 0.8 were considered as small, medium, and large, respectively.

We selected independent variables in two of the steps [54]. In the first step (filter method [54]), we chose parameters from the sensor-derived parameters that had a *p*-value less than 0.05 and a *d* ≥ 0.4. In the second step (embedded method [54]), these independent predictors were fed to a model in order to discriminate the pre-frail from the two other groups (non-frail and frail). The Receiver Operating Characteristic (ROC) curve, performance (sensitivity, specificity, and accuracy), and area under the curve (AUC), were calculated based on the one-vs-rest method [55]. Of the independent predictors, those with an AUC greater than 0.7 were used to develop discrimination models. To select the independent predictors, the whole dataset was used [56,57].

We developed four models as follows: (1) the step model: using step parameters, such as the total number of steps; (2) the physical activity pattern (PAP) model: PAP parameters such as the total walking and postures duration; (3) the physical activity behavior (PAB) model: PAB parameters, such as sedentary; and (4) the combined model: all of the parameters such as total number of steps, total walking, and sedentary. To train and test the model, we used a k-fold cross validation (k = 5). In this method, the dataset was randomly partitioned into five subsamples [58,59]. Four partitions were used to train each model and one partition, which was not used for training, was used for validating each model. This step performed for five times. The average and standard deviation of the performance parameters for the validation phase were reported. The performance parameters that were measured for each model were sensitivity, specificity, accuracy, and the AUC [59].

## 3. Results

### 3.1. Demographic and Clinical Characteristics

Originally, 163 participants had consented to participant in this study. Data from 10 participants was excluded because of low wear-time (*n* = 3), less than two days of recording (*n* = 5), and forgetting to put on the sensor (*n* = 2). The remaining 153 participants (75 ± 10 years and 79% female) were included in the study, where 42 (27%) were considered as non-frail, 78 (51%) pre-frail, and 33 (22%) frail (Table 1). In the progression of the frailty status among the participants, we observed a trend in several demographic characteristics, such as BMI, depression, fear of falling, cognitive dysfunction, number of the prescribed medication, and number of comorbidities. The pre-frail group had a significantly higher BMI than the non-frail group (*p*-value ≤ 0.001). Depression in the frail group was significantly higher than in the pre-frail group (*p*-value = 0.002). A fear of falling in the pre-frail group was lower than that in the frail group (*p*-value = 0.006) (Table 2).

### 3.2. Sleep Quantity Parameters

In the sleep parameters, we observed a trend of reduction in TiB and TST, and a trend of increase in SOL in the progression of frailty. Specifically, TiB (*p*-value = 0.010, *d* = 0.50) and TST (*p*-value = 0.027, *d* = 0.45) differed significantly in non-frail and pre-frail groups (Table 2). Interestingly, the sleep side position (*p*-value = 0.001, *d* = 0.65) was significantly different in the pre-frail and frail group. No sleep quantity parameters were capable of discriminating between the three groups of frailty statuses.

### 3.3. Physical Activity Pattern Parameters

In the physical activity pattern parameters, we observed a trend of reduction in standing and walking, and a trend of increase in the lying duration (Table 2). Specifically, the standing duration was significantly different between the pre-frail and non-frail (*p*-value = 0.003, *d* = 0.57). The total duration of walking, longest unbroken walking bout, and the median walking bout were capable of discriminating between the comparisons group of groups. When each parameter was fed into the model in order to identify the pre-frail group, only the total walking duration and longest unbroken walking bout had an AUC of >0.7, while the median walking bout showed an AUC of <0.7, and specificity, less than 50% (Table 3).

### 3.4. Stepping Parameters

All of the stepping parameters showed a trend of decline by frailty progression (Table 2). The total number of steps and the longest unbroken stepping bout were significantly different between the non-frail vs. pre-frail, and the pre-frail vs. frail groups, and they showed a significant independent predictor with an AUC > 0.7 for pre-frail status (Table 3). The median stepping bout was not significant between the groups and was also rejected when it was independently fed to the model, for an AUC < 0.7 (Table 3).

### 3.5. Physical Activity Behavior Parameters

In the overall physical activity behavior parameters, we observed a reduction trend (from non-frail to frail) in the duration of light activity and moderate-to-vigorous activity, and a trend of increase in sedentary behavior (Table 2). Specifically, the percentage of sedentary behavior (*p*-value < 0.001, *d* = 0.98), duration of light activity (*p*-value = 0.001, *d* = 0.62), percentage of light activity (*p*-value < 0.001, *d* = 0.79), and percentage of MtV activity (*p*-value < 0.001, *d* = 1.13), differed significantly between the non-frail and pre-frail groups. Among the parameters, the total duration of sedentary behavior, median light activity, and total duration of MtV, differed significantly between the groups. The median light activity had a very low specificity and AUC; therefore, it was not considered for building the model so as to discriminate the pre-frail from other groups. However, the total sedentary and MtV was used in building this model. Also, we observed a trend of reduction in the percentage of participants in each group who met the physical activity recommendation from the HHS. The odds of meeting the HHS guidelines in the non-frail and pre-frail groups varied significantly (*p*-value < 0.001)

### 3.6. Performance of Models for Discriminating Pre-Frail Status

Among the non-combined models, the stepping model and the physical activity pattern (PAP) model had the same level of high sensitivity (88.6%), while the specificity of physical activity behavior (PAB) was the highest (77.9%). The accuracy of PAB and PAP were slightly (less than 2%) higher than the stepping model (Table 4). Overall, the four models showed a large AUC of ≥0.8 (Table 4). The combined model was a composite of all of the sensory parameters that were independently predictive of pre-frail status (see Table 3). This combined model had the highest sensitivity, specificity, accuracy, and AUC (91.8%, 81.4%, 84.7%, and 0.88, respectively) for identifying the pre-frail status (Table 4).

## 4. Discussion

This study examined the association between the measurable physical activities, from a pendant accelerometer-based sensor, and the different frailty stages. Prior frailty studies, which had used sensor-derived parameters, were often based on the supervised assessment of motor performances (e.g., gait assessment, balance, Timed Up & Go, etc. [24,60,61,62]), which were unsuitable for the remote monitoring of the frailty stages. There were few studies that attempted to determine the frailty stages based on activity monitoring [32]. However, to our knowledge, none of the prior studies took into account both the daytime and nighttime (e.g., sleep) activities in order to distinguish the pre-frailty stage. The current study used and determined the most sensitive and independent metrics that were measurable from a single pendant sensor, including the physical activity pattern/stepping, physical activity behaviors, and sleep parameters, in order to discriminate among the frailty categories in community-dwelling older adults. Furthermore, we examined which activity-derived parameters were the most sensitive in order to distinguish pre-frailty, which was known as a potentially reversible frailty stage [35,36,37]. From a model construction standpoint, we not only used uni-variate, multi-variable analysis, and embedded feature selections, but we also applied a decision trees model, which had been shown to be a more robust model than conventional multi-variable models (e.g., the linear regression of logistic regression model) [62,63]. Together, the proposed approach allowed for distinguishing the pre-frailty stage from the other stages during activities of daily living, via a simple and practical wearable platform. More specifically, the results suggested that the most sensitive descriptors of the pre-frailty stage were total sedentary duration, total moderate-to-vigorous activity duration, total walking duration as a percentage of 24 h activities, longest unbroken walking bout, total daily steps, and longest unbroken steps.

While several instruments were proposed for assessing frailty (e.g., the frailty index, proposed by Rockwood et al. [11], and the frailty phenotypes, proposed by Fried et al. [10]), they were unsuitable for in-place and remote monitoring of the frailty stages, because they often required a supervised administration of the test, relied on subjective or semi-objective data obtained from self-reported inactivity and/or availability of patient health records, and were often insensitive to change over time [26,64]. The proposed model/platform and its practical form factor (using a pendant instead of securing a sensor to the chest) might have addressed these limitations and thus could have facilitated the development of a telehealth platform, based on wearables and activity monitoring. Most importantly, the results of this study suggested that a single pendant sensor could distinguish the pre-frail stage from other frailty stages. In addition, we previously demonstrated that two days of activity monitoring would be enough to determine the frailty stages [51]. This in turn, might have allowed for the tracking of changes in the frailty stages, with a relatively high time resolution (48 h), which would have provided a window of opportunity for timely preventive or therapeutic interventions that might have delayed the progression of frailty and identifying modifiable factors. This might have contributed to the deteriorating resilience (e.g., medication adverse effect, depression, immobility, etc.). 

Our results were in agreement with previous studies, which suggested that total number of steps, amount of sedentary behaviors, and moderate-to-vigorous activity were associated with the progression of the frailty stages [31,32,60,61]. However, to our knowledge, this was the first study that integrated a greater variety of sensor-based measurable physical activity metrics, including steps, sleep, activity pattern, and activity behavior, into a cohesive model in order to determine the independent descriptors of the frailty stages. In addition, our study was able to demonstrate which activity related parameters, which were measurable by a pendant sensor, allowed for determining the pre-frailty stage. Our results suggested that in order to more accurately discriminate between the pre-frail and non-frail stages, a more comprehensive set of measurable physical activity categories, including sleep, activity pattern, stepping parameters, and activity patterns, could enable a significant discrimination, with effect sizes ranging from medium to large. The largest effect sizes were observed for the total walk duration, as a percentage of 24 h activities; total daily number of steps; and MtV behavior (Cohen’s effect, size *d* > 1.00). The discrimination between the pre-frail and frail was, however, more challenging. Nevertheless, the moderate effect sizes were observed when the total walk, total step, longest unbroken steps number, median light bout activity, or total MtV activities were considered (*d* > 0.50). Using the univariate analysis, none of the sleep parameters were enabled to simultaneously distinguish the pre-frail from other groups, and thus were excluded from the model design. Among the remaining parameters, the most sensitive parameters were the total sedentary duration, total MtV duration, and total walk duration, which were able to identify the pre-frail from the other groups with an AUC of greater than 0.90. 

Overall, we found that the frail group had the highest sedentary behaviors, which was an indicator of functional disability, as was reported in previous studies [65]. Furthermore, as previous literature had mentioned, we observed that the frail group had the highest sedentary duration, which might have led to a higher comorbidity [66]. The HSS guidelines emphasized the importance of meeting the physical activity requirements, namely, having more than 300 min per week of moderate-to-vigorous activity. We observed that the odds of meeting the guideline recommendation were significantly lower in the frail group, which might have increased the risk of adverse health outcomes [67,68].

Further investigation would be needed into the association between frailty status and light activity, which included domestic chores like instrumented activity of daily living (e.g., cooking, household tasks, etc.). In our study, light activity was unable to discriminate between the frail and pre-frail, but it did enable the distinguishing of the pre-frail from non-frail stage. A study on older females with Parkinson’s disease reported an association between light activity duration and cognitive dysfunction [69]. Thus, light activity might have been representative of instrumental activities of daily living or cognitive function. On the other hand, recent studies suggested that the combination of frailty and cognitive impairment (cognitive frailty) could have better determined the prospective decline in motor and cognitive performance [70,71,72,73]. Our study did not incorporate cognitive function into the model, because it was based on the Fried frailty phenotypes, which did not include cognitive performance. Thus, further exploration would be warranted to better understand the association between light activity and frailty phenotype progression, mediated by measures of cognitive function and changes in cognitive function. Indeed, future studies investigating sensory-derived data as measures for cognitive function that integrate physical performance-based models (as presented in the current study) could provide a more holistic understanding of the progression of the frailty stages in older adults.

We observed a reduction in nocturnal sleep parameters, such as total sleep time and time in bed, and an increase in sleep onset latency in the advancing frailty stages. The same observation was reported in a previous cohort of older community-dwelling men (*n* = 3133), where the odds of sleep disturbances had increased by the risk of frailty [34]. In our study, the non-frail group had significantly lower sleep disturbances, but group comparison between the pre-frail and frail did not achieve a statistically significant level in our sample.

Finally, in order to examine the robustness of a predictive multiple variables model, so as to identify the pre-frail group among other groups, we used k-fold cross validation (k = 5) method, in which a 20% randomly selected dataset were used for the validation of the model. Using this approach, namely, stepping; the physical activity pattern; and physical activity behavior models were able to distinguish between the pre-frail from the others groups, with an AUC of 0.87, 0.85, and 0.85, respectively. The combination model improved on the discriminative power, with an AUC of 0.88.

To improve the level of comfort and mimic the telehealth platforms, which often incorporated a pendant sensor (e.g., personal emergency response system [PERS], such as pendant automatic fall detectors), we used a pendant accelerometer to monitor sleep and activities instead of securing the sensor on the chest, which had been used in previous studies [41,43]. This approach might have affected the accuracy of the activity detection, as well as the estimation of the sleep parameters of interest. Despite this potential limitation, the measured parameters achieved a statistically significant level so as to distinguish the pre-frailty stages, thus creating a more realistic sensor-based method in order to monitor the frailty stages and their fluctuation over time, without hindering the everyday activities of daily living. In addition, the proposed study design could have facilitated the integration of the designed model in the currently available pendant PERS platforms. 

## 5. Limitations

This study had several limitations. The sample size (*n* = 163) was relatively small and may be insufficient to represent the general older adults population. In addition, the feature selection was done based on the entire sample, and the sample size might have been insufficient for the purpose of the k-fold cross validation model. However, as recommended by the prior literature, this approach was shown to be more robust than the conventional approaches for relatively small sample size studies [56,57].To better examine the validity and reliability of the proposed model, another study was needed to confirm that the results remained the same when using an independent and larger dataset. Therefore, the results needed to be confirmed in a larger sample, in order to be generalized. As this was a cross-sectional study, the sensitivity to change over time for the proposed model was unclear and needed to be verified in another study. In addition, the ability of the proposed model to predict the prospective adverse health outcomes, including mortality or loss of independency, should have been examined in another study. We used the Fried physical phenotypes criteria to determine the frailty stages, which carried some limitations, including a lack of consideration for cognitive function and using the categorical stages (non-frail, pre-frail, and frail) instead of a continuous scale. Fine tuning the model outputs in comparison with other well-established frailty assessment tools, such as the frailty index (an alternative frailty conceptual model that measures accumulation of deficits and provides a continuous scale instead of categorical), might have been useful for designing a more sensitive to change metric for the purpose of longitudinal studies. Two days of continuous monitoring (48 h) might not have been sufficient in order to represent the overall in-place activities of older adults. However, as suggested in our previous study [51], two days of continuous monitoring yielded a reliable representation of daily physical activities in a geriatric population, in particular among those with the frailty status, because of the reduction in the activities complexities or day-to-day variation, as suggested by previous studies [74,75,76]. On the other hand, in order to determine the causal factors that might have led to physical frailty, for instance in response to medication, a high time resolution, to determine frailty phenotypes, might have been considered as an advantage of the proposed approach. However, future studies were needed that would examine whether the proposed frailty model was sensitive to change and could track changes in the frailty stages over time.

## 6. Conclusions

We demonstrated that a single pendant accelerometer enables determining the frailty stages, including pre-frailty, via an in-place monitoring of the spontaneous daily physical activity, including the day time and night time. Among the measurable parameters, using a single pendant accelerometer-based device, a combination of step parameters (e.g., number of daily taken steps, longest unbroken steps), activity behavior (e.g., moderate-to-vigorous and sedentary activities), and postures (e.g., duration of standing, walking, and longest unbroken walking bout duration) enables the distinguishing of the pre-frailty stage among non-frail and frail stages, with AUC of 0.88. The proposed model and the form factor of the sensor that was used (pendant instead of securing sensor to the skin) provide advantages, compared with the conventional frailty assessment tools, for the purpose of in-place and prolonged screening (over days and months). In addition, it doesn’t require a supervised administration of testing (unsupervised monitoring of frailty stages); it is objective; and does not need patient health records, demographics, or self-report, which makes it easy and cost-effective for deployment for in-place monitoring platforms. It can also facilitate in the development of a telehealth platform, based on wearable technology, to determine the modifiable factors that are significant for the advancing frailty stages (e.g., use of medication, which may negatively impact subject resilient; sleep deprivation; depression; cognitive decline; etc.). These potential applications, however, need to be validated in future studies.

## Figures and Tables

**Table 1:** Demographic and clinical characteristics reported by mean ± standard deviation.

Parameter	Non-Frail (N)	Pre-Frail (P)	Frail (F)	N vs. P (*p*-Value)	P vs. F (*p*-Value)
Number of participants	42	78	33		
Age, years	74.02 ± 7.37	75.25 ± 11.53	78.03 ± 11.20	0.527	0.191
BMI, kg/m^2^	25.21 ± 5.64	29.76 ± 6.88	31.18 ± 7.01	0.000	0.289
Depression (CES-D)	6.62 ± 5.64	8.75 ± 7.14	12.89 ± 6.33	0.083	0.002
Concern for fall (SFES-I)	20.69 ± 4.22	23.43 ± 11.14	29.69 ± 15.89	0.182	0.006
Gender (female), %	82%	54%	51%	0.001	0.247
Number of prescribed medication, N	2.5 ± 1.8	4.1 ± 3.8	6.0 ± 3.4	0.530	0.520
Number of over the counter medication, N	3.0 ± 2.5	2.7 ± 2.2	2.5 ± 2.5	0.150	0.080
Number of comorbidities, N	2.3 ± 1.8	3.7 ± 2.2	4.7 ± 1.7	0.710	0.490
Fall history:					
0 falls	71%	69%	69%	0.177	0.425
1–3 falls	24%	30%	23%	0.090	0.800
>3 falls	4%	1%	9%	0.368	0.020

BMI—body mass index; CES-D—Center for Epidemiologic Studies Depression; FES-I—Fall Efficacy Scale-International.

**Table 2:** Results of sensor derived parameters assessment, stratified by frailty status.

Parameters	Non-Frail (N) †	Pre-Frail (P) †	Frail (F) †	*p*-Value (Effect Size ‡)	
				N vs. P	P vs. F
**Sleep Quantity**					
Time in bed, min	494.3 ± 114.4	434.7 ± 125.3	402.4 ± 127.6	0.010 (0.50)	0.209 (0.26)
Sleep onset latency, min	16.9 ± 7.5	18.5 ± 7.9	20.0 ± 8.4	0.290 (0.20)	0.369 (0.18)
Total sleep time, min	367.5 ± 86.2	321.9 ± 116.5	300.9 ± 119.4	0.027 (0.45)	0.360 (0.18)
Wake after sleep onset, min	103.7 ± 48.0	89.4 ± 41.7	75.5 ± 43.0	0.081 (0.32)	0.129 (0.33)
Sleep efficiency, %	78.4 ± 9.3	77.5 ± 10.9	79.6 ± 11.6	0.650 (0.09)	0.333 (0.19)
Sleep supine position, %	43.6 ± 21.0	41.2 ± 23.2	47.5 ± 31.4	0.594 (0.11)	0.222 (0.23)
Sleep prone position, %	14.7 ± 17.5	12.3 ± 16.7	18.8 ± 23.3	0.484 (0.14)	0.091 (0.32)
Sleep side position, %	34.6 ± 17.1	34.6 ± 24.2	19.7 ± 21.1	0.994 (0.00)	0.001 (0.65)
**Physical Activity Pattern**					
Total sit, %	43.3 ± 15.6	46.9 ± 17.1	45.7 ± 17.1	0.253 (0.22)	0.734 (0.07)
Total stand, %	16.9 ± 5.8	13.6 ± 5.8	11.3 ± 5.7	0.003 (0.57)	0.060 (0.40)
Total walk, % *	8.7 ± 3.9	5.1 ± 3.3	3.2 ± 3.2	0.000 (1.02)	0.012 (0.57)
Total Lye, %	30.9 ± 15.6	34.3 ± 20.4	39.7 ± 20.9	0.348 (0.19)	0.188 (0.26)
Longest unbroken sitting bout, s	5356.7 ± 3499.1	5351.3 ± 3042.0	5576.5 ± 3927.4	0.993 (0.00)	0.749 (0.06)
Median sitting bout, s	96.2 ± 72.9	89.6 ± 105.2	81.1 ± 92.0	0.712 (0.07)	0.666 (0.09)
Longest unbroken standing bout, s	385.3 ± 387.3	550.6 ± 723.8	553.2 ± 360.5	0.132 (0.28)	0.983 (0.00)
Median standing bout, s	15.0 ± 3.2	13.3 ± 7.5	13.3 ± 11.7	0.247 (0.29)	0.983 (0.00)
Longest unbroken walking bout, s *	351.3 ± 347.9	187.9 ± 223.9	110.3 ± 132.4	0.001 (0.56)	0.002 (0.42)
Median walking bout, s *	9.7 ± 1.0	9.0 ± 1.5	8.3 ± 1.9	0.020 (0.51)	0.018 (0.43)
**Stepping Parameters**					
Total step, N/1000 *	12.2 ± 6.1	6.7 ± 4.2	4.3 ± 4.3	0.000 (1.04)	0.018 (0.57)
Longest unbroken stepping bout, N *	694.3 ± 743.0	322.9 ± 411.0	162.5 ± 184.2	0.000 (0.62)	0.006 (0.57)
Median stepping bout, N	13.5 ± 2.2	10.8 ± 4.7	8.8 ± 5.2	0.001 (0.75)	0.113 (0.38)
**Physical Activity Behavior**					
Median Sedentary bout, s	323.8 ± 2044.6	491.1 ± 4243.4	29.7 ± 16.7	0.783 (0.05)	0.497 (0.15)
Total sedentary, h *	9.6 ± 2.6	11.7 ± 3.2	13.2 ± 4.2	0.001 (0.73)	0.029 (0.40)
Total sedentary, %	70.4 ± 12.7	81.1 ± 8.9	84.9 ± 7.0	0.000 (0.98)	0.066 (0.47)
Median light bout, s *	10.8 ± 2.2	9.6 ± 2.8	8.3 ± 2.5	0.011 (0.50)	0.013 (0.51)
Total light, h	3.2 ± 1.3	2.4 ± 1.2	2.1 ± 0.9	0.001 (0.62)	0.206 (0.29)
Total light, prc	23.7 ± 9.9	16.7 ± 7.7	13.9 ± 6.2	0.000 (0.79)	0.105 (0.39)
Median MtV, s	6.8 ± 1.9	6.8 ± 2.4	6.3 ± 1.8	0.990 (0.00)	0.267 (0.24)
Total MtV, min *	47.7 ± 30.7	19.6.3 ± 20.5	11.2 ± 14.6	0.000 (1.08)	0.047 (0.47)
Total MtV, % *	6.0 ± 4.0	2.2 ± 2.4	1.2 ± 1.5	0.000 (1.13)	0.066 (0.50)
HHS guideline, % (N)	50.0	16.0	3.1	0.000 (-)	0.109 (-)

†—average ± standard deviation; ‡—effect size calculated based on Cohen’s *d* effect size for normal distribution (*d*) or for those who reject the normality (*r*), *—parameters that *p*-value < 0.05 and *d/r* ≥ 0.4; MtV—moderate-to-vigorous activity; bout—consecutive interval of a physical activity behavior without interrupt; HHS guideline—U.S. Department of Health and Human Services recommended guideline for the MtV duration.

**Table 3:** The performance of each parameter to discriminate the pre-frail group from the non-frail and frail groups.

	Sensitivity †, %	Specificity †, %	Accuracy †, %	AUC
**Physical activity behaviors**				
Total sedentary, h *	91.9 ± 3.8	66.7 ± 4.4	76.3 ± 1.9	0.91 ± 0.03
Median light bout, s	100.0 ± 0.0	0.0 ± 0.0	51.9 ± 0.2	0.50 ± 0.00
Total MtV, min *	85.4 ± 3.3	77.8 ± 4.9	78.8 ± 1.7	0.92 ± 0.02
**physical activity patterns**				
Total walk, % *	86.1 ± 4.5	73.7 ± 6.7	78.0 ± 1.9	0.90 ± 0.02
Longest unbroken walking bout, s *	84.2 ± 5.6	67.9 ± 6.8	73.0 ± 1.6	0.87 ± 0.02
Median walking bout, s	85.3 ± 8.1	32.4 ± 13.2	59.0 ± 2.2	0.64 ± 0.02
**stepping parameters**				
Total step, N/1000 *	88.9 ± 3.0	74.0 ± 3.4	78.7 ± 1.2	0.89 ± 0.02
Longest unbroken stepping bout, N *	85.0 ± 5.7	75.8 ± 4.3	75.3 ± 1.9	0.88 ± 0.03

†—average ± standard deviation; AUC—area under the curve; *—parameters with asterisks were used later to develop the model.

**Table 4:** The performance of models to separate the pre-frail group from the rest of the groups (non-frail and frail).

Models	Sensitivity †, %	Specificity †, %	Accuracy †, %	AUC †
Step Model	88.6 ± 3.8	77.5 ± 1.3	79.7 ± 0.5	0.87 ± 0.02
Physical Activity Pattern Model	88.6 ± 3.3	74.2 ± 1.7	80.6 ± 0.4	0.85 ± 0.02
Physical Activity Behavior Model	87.1 ± 6.1	77.9 ± 2.0	80.4 ± 0.6	0.85 ± 0.03
Combined Model	91.8 ± 4.2	81.4 ± 2.2	84.7 ± 0.4	0.88 ± 0.03

AUC—area under the curve; †—the mean ± standard deviation reported for the validation datasets based on a 5-fold cross validation; Step Model—the total number of steps and longest unbroken stepping bout; Physical Activity Pattern Model—the total walk and longest unbroken walking bout; Physical Activity Behavior Model—the total steps and longest unbroken stepping bout; Combined Model—all of the mentioned parameters.
